# Immune cell score in pancreatic cancer—comparison of hotspot and whole-section techniques

**DOI:** 10.1007/s00428-019-02549-1

**Published:** 2019-03-07

**Authors:** Kyösti Tahkola, Joni Leppänen, Maarit Ahtiainen, Juha Väyrynen, Kirsi-Maria Haapasaari, Tuomo Karttunen, Ilmo Kellokumpu, Olli Helminen, Jan Böhm

**Affiliations:** 1 Department of Surgery, Central Finland Central Hospital, Jyvaskyla, Finland; 2 Department of Gastroenterology and Alimentary Tract Surgery, Tampere University Hospital, Tampere, Finland; 3 Cancer and Translational Medicine Research Unit, Medical Research Center Oulu, University of Oulu and Oulu University Hospital, Oulu, Finland; 4 Department of Education and Research, Central Finland Central Hospital and University of Eastern Finland, Jyvaskyla, Finland; 5 Department of Oncologic Pathology, Dana-Farber Cancer Institute and Harvard Medical School, Boston, MA USA; 6 Department of Pathology, Central Finland Central Hospital, Jyvaskyla, Finland

**Keywords:** Immune cell score, Pancreatic cancer, Whole section, Microenvironment, CD3, CD8

## Abstract

An immune cell score (ICS) was introduced for predicting survival in pancreatic ductal adenocarcinoma (PDAC). Few studies have compared different methods of evaluating immune infiltrate. This study compared ICSs determined in whole sections or tissue microarray-like hotspots for predicting survival after PDAC surgery. We included in 79 consecutive patients from a single geographical area that underwent surgery for PDAC (R0/R1, stages I–III). We performed digital image analyses to evaluate CD3 and CD8 staining. ICSs were classified as low, moderate, or high, based on the numbers of immune cells in the tumour core and invasive margin. We compared ICS groups determined with the hotspot and whole-section techniques. Associations between ICS and survival were analysed with Cox regression models, adjusted for sex, age, tumour stage, differentiation grade, perineural invasion, and resection radicality. In hotspot ICS analysis, 5-year overall survival rates for low, moderate, and high groups were 12.1%, 26.3%, and 26.8%, respectively (*p* = 0.193). In whole-section analyses, overall survival rates were 5.3%, 26.4%, and 43.8%, respectively (*p* = 0.030). In the adjusted Cox model, whole-section ICS groups were inversely associated with the overall mortality hazard ratio (HR): low, moderate, and high ICS groups had HRs of 1.00, 0.42 (95% CI 0.20–0.88), and 0.27 (95% CI 0.11–0.67), respectively. The number of immune cells per square millimetre in the tumour core and the invasive margin were significantly higher and had a wider range in hotspots than in whole-tissue sections. Accordingly, ICS could predict survival in patients with PDAC after surgery. Whole tissue section ICSs exhibited better prognostic value than hotspot ICSs.

## Introduction

Cancer progression is known to be strongly influenced by the host immune response, which is represented by immune cell infiltrates [1, 2]. Multiple scoring systems have been developed to evaluate the association between the host immune response and survival of patients with cancer [3–6]. Traditional tumour node metastasis (TNM) staging is the gold standard for evaluating prognosis in most solid tumours, and TNM staging was recently updated by the American Joint Committee on Cancer (AJCC) in the 8th edition of the staging manual [7]. However, TNM staging ignores the impact of the host immune response. Nevertheless, it has been shown that tumour-infiltrating lymphocytes were correlated with survival in rectal, oesophageal, and gastric cancers [8–10]. Indeed, in colorectal cancer, a computer-assisted evaluation of the quantity of CD3^+^ and CD8^+^ lymphocytes, known as the Immunoscore®, was found to be a reproducible, independent prognostic parameter [11]. Thus, it was suggested that integrating the TNM and Immunoscore® might provide more accurate staging.

Pancreatic ductal adenocarcinoma (PDAC) is the seventh deadliest cancer worldwide [12]. It is typically diagnosed in the late stages, which rules out curative surgery [13]. PDAC is characterised by a vast stromal reaction, inflammatory response, and neovascularization, which all contribute to resistance against anti-cancer drugs [14]. Even with surgery, survival rates remain low [15]. Inflammatory response and tumour microenvironment have been researched in recent years, and their significance in pancreatic cancer is becoming evident [16]. Various biomarkers have been investigated to enhance determinations of prognosis and to find the most suitable therapeutic approaches [17]. Although various combinations of intra-tumoural and peri-tumoural immune cells have been proposed as prognostic factors in pancreatic cancer [5], complete knowledge is lacking, due to the complex interplay between chronic inflammation and the immune response.

We recently introduced a T lymphocyte-based immune cell score (ICS), which we applied to pancreatic ductal adenocarcinoma (PDAC). This ICS showed good correlation with survival [18]. That study was based on tissue microarrays (TMAs), which are widely used in research to facilitate investigations of specific characteristics in a large number of tissue samples. Nevertheless, the TMA technique has a well-known risk of sampling error. Investigators have attempted to avoid this problem by acquiring multiple tissue cores from several hotspots. However, very few studies have compared TMA and whole-tissue techniques for evaluating immune cell infiltrates.

The primary aim of the present study was to test the prognostic significance of ICS in a separate consecutive series of patients with PDAC in Northern Finland. The secondary aim was to compare the TMA-like hotspot technique and whole-tissue section technique for efficacy in an ICS analysis. This study was designed and performed according to reporting recommendations for tumour marker prognostic studies [19].

## Materials and methods

### Patients

This retrospective cohort study included paraffin-embedded archival specimens of 95 consecutive patients with PDAC that received surgical treatment in 1993–2015 at Oulu University Hospital. Patients were excluded when they had advanced disease or distant metastases (stages 3–4, according to TNM, 7th edition, which was in use at the time of patient selection) or R2 resection margins. The final series consisted of 79 patients with stages 1-2B (TNM 7th edition) and R0/R1 tumours. During the present study, we re-staged the specimens, according to the TNM, 8th edition, which resulted in the distribution of stages described in Table 1. The histological diagnoses were confirmed by an expert gastrointestinal pathologist. All patients underwent either a pancreaticoduodenectomy, according to Whipple (*n* = 72), or a total pancreatectomy (*n* = 7). No patient received neoadjuvant therapy. The mean patient age at diagnosis was 64 years (SD 9.3). Patient characteristics are described in Table 1.

**Table 1:** Clinical characteristics and immune cell score based on whole sections

Variables	Total	Immune cell score 0–1	Immune cell score 2	Immune cell score 3–4	*p* value
	*N* = 79	*N* = 39	*n* = 22	*n* = 18	
Sex					
Male	*n* = 41	*n* = 18	*n* = 10	*n* = 13	0.145
Female	*n* = 38	*n* = 21	*n* = 12	*n* = 5	
Age					
< 65	*n* = 36	*n* = 18	*n* = 10	*n* = 8	1.000
65–75	*n* = 35	*n* = 17	*n* = 10	*n* = 8	
> 75	*n* = 8	*n* = 4	*n* = 2	*n* = 2	
Tumour					
T1a	*n* = 0	*n* = 0	*n* = 0	*n* = 0	0.096
T1b	*n* = 5	*n* = 2	*n* = 1	*n* = 2	
T1c	*n* = 14	*n* = 4	*n* = 8	*n* = 2	
T2	*n* = 43	*n* = 21	*n* = 10	*n* = 12	
T3	*n* = 17	*n* = 12	*n* = 3	*n* = 2	
Node					
N0	*n* = 36	*n* = 20	*n* = 11	*n* = 5	0.361
N1	*n* = 29	*n* = 12	*n* = 9	*n* = 8	
N2	*n* = 14	*n* = 7	*n* = 2	*n* = 5	
Stage					
IA	*n* = 8	*n* = 2	*n* = 4	*n* = 2	
IB	*n* = 17	*n* = 10	*n* = 5	*n* = 2	
IIA	*n* = 11	*n* = 8	*n* = 2	*n* = 1	0.356
IIB	*n* = 29	*n* = 12	*n* = 9	*n* = 8	
III	*n* = 14	*n* = 7	*n* = 2	*n* = 5	
Grade					
I	*n* = 15	*n* = 6	*n* = 7	*n* = 2	0.473
II	*n* = 29	*n* = 13	*n* = 9	*n* = 7	
III	*n* = 17	*n* = 10	*n* = 2	*n* = 5	
Perineural invasion					
Negative	*n* = 42	*n* = 22	*n* = 10	*n* = 10	0.694
Positive	*n* = 37	*n* = 17	*n* = 12	*n* = 8	
Resection					
R0	*n* = 50	*n* = 22	*n* = 15	*n* = 13	0.441
R1	*n* = 29	*n* = 17	*n* = 7	*n* = 5	
Disease-specific survival (%)					
1 year	72.0	61.0	90.9	69.5	**0.020**
3 years	27.5	10.9	39.6	55.6	
5 years	19.5	5.4	26.4	55.6	
Overall survival (%)					
1 year	70.0	59.1	90.9	65.7	
3 years	25.3	10.5	39.6	43.8	**0.030**
5 years	17.9	5.3	26.4	43.8	

*P*-values showing significant association (< 0.05) are marked in bold

The clinical data were obtained from patient records and patient survival data. Cause of death was obtained from the Cause of Death Registry from Statistics Finland. Our use of the samples and patient data were approved by the Oulu University Hospital Ethics Committee and by the National Authority for Medicolegal Affairs (VALVIRA).

### Histopathological examination

Histopathological reviews of tumour specimens were performed by two experienced gastrointestinal pathologists (TK, JB). Tumour stage was determined according to the 7th edition of the UICC/AJCC TNM categories, which were current at the time of patient selection, and re-staging was performed according to the 8th edition, later during the study.

### Immunohistochemistry

Tissue sections (3-μm thick) from a representative tumour tissue block were immunohistochemically stained with anti-CD3 antibodies (Novocastra, NCL-L-CD3-565, clone LN10, 1:50) and anti-CD8 antibodies (Novocastra, NCL-L-CD8, Clone 4B11, 1:200). Antigen retrieval was performed with tris-EDTA buffer at pH 9 in a microwave at 98 °C for 15 min. Samples were incubated with diluted antibodies at room temperature for 30 min. Bound antibodies were detected with the Dako Envision Kit (DAKO, Copenhagen, Denmark). DAB was used as a chromogen, and haematoxylin was used as a counterstain.

### Immune cell score determination

**Fig. 1:**
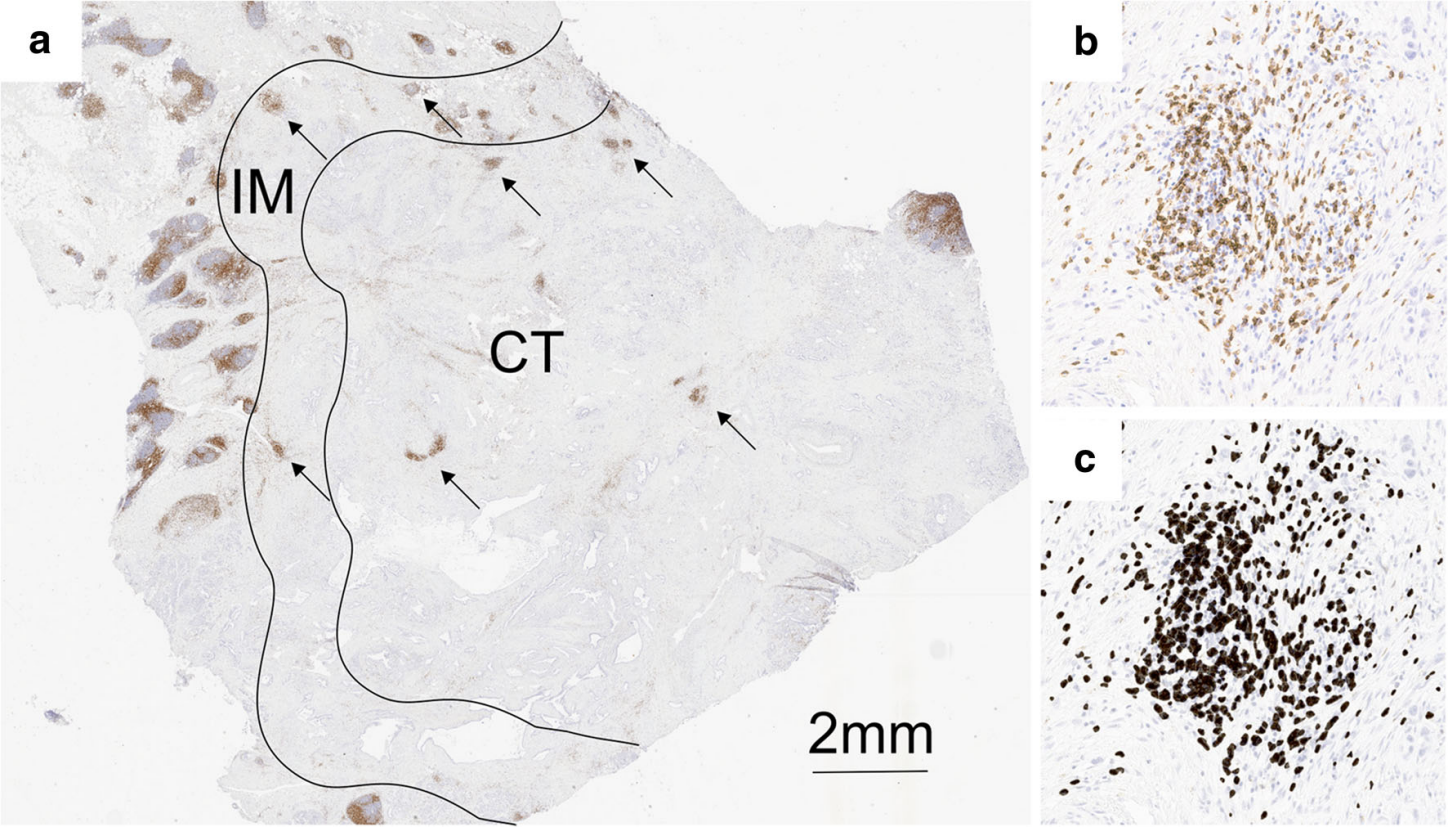
T cell infiltration in different regions of pancreatic ductal adenocarcinoma. **a** Image of a whole section of anti-CD3-stained tissue displaying the centre of the tumour (CT) and the invasive margin (IM). The highest densities of CD3^+^ T cells were found in lymphoid aggregates, localised in the IM or the CT (arrows). **b** A hotspot in the CT that contains a lymphoid aggregate shows numerous positive T cells and fewer scattered tumour cells. The hotspot T cell density was frequently much higher than the average whole-section T cell density. **c** The corresponding image analysis shows the counted cells (dark grey shading)

Immunohistochemical stains were assessed without knowledge of the clinical data. CD3^+^ and CD8^+^ cells were assessed with digital image analysis. Stained whole-tissue sections were scanned with an Aperio digital slide scanner AT2 Console (Leica Biosystems Imaging Inc., Nussloch, Germany), then analysed with the ImageJ program and a previously validated cell counting method [20]. Immune cell hotspot areas (0.28 mm^2^) were defined digitally according to an ICS protocol, in both the tumour area and the invasive margin, which simulated the original TMA technique [18]. As previously described, the selected hotspot areas were both representative of the tumour and rich in immune cells. Tertiary lymphoid structures were not included in the hotspots. The invasive margin was defined as a 0.5-mm-wide region on each side of the tumour, which included cells at the border between the tumour cells and normal pancreatic tissue. CD3^+^ and CD8^+^ cells were counted separately in hotspot areas, in the whole tumour area, and in the invasive margin area (Fig. 1). Samples were divided into two groups of “high” and “low” cell densities, based on the calculated numbers of positively stained lymphocytes (cells/mm^2^).

To determine the cut-off values for an ICS with optimal sensitivity and specificity, we used receiver operating characteristic (ROC) curves for each group, based on disease-specific 3-year mortality. The cut-off values for the hotspot counts were, as follows (cells/mm^2^): 1116 for CD3^+^ in the tumour core, 1314 for CD3^+^ in the invasive margin, 1185 for CD8^+^ in the tumour core, and 998 for CD8^+^ in the invasive margin. The cut-off values for the whole-section counts were 396, 370, 120, and 157, respectively.

The high and low groups were used to construct ICS groups, which ranged from ICS 0 (low CD3^+^ and CD8^+^ densities in both regions) to ICS 4 (high CD3^+^ and CD8^+^ densities in both regions), as described previously [18]. According to the ICS protocol, three groups were formed (high, moderate, and low).

### Statistical analysis

We used the chi-square test to calculate differences in clinicopathological variables between groups. We used the Kaplan–Meier method and log-rank test to evaluate disease-specific survival (DSS) and overall survival (OS). Survival times were calculated from the date of surgery to the time of death or the end of follow-up (December 31, 2015). We performed univariate and multivariate Cox proportional hazards regression models to calculate hazard ratios for DSS and OS. These models were adjusted with the following a-priori determined confounders: age, sex, tumour stage (according to the 8th edition of the UICC/AJCC TNM categories), differentiation grade, and resection radicality (R0/R1). *P* values less than 0.05 were considered significant. The statistical analysis was performed with IBM SPSS statistics 24 for Windows (IBM Corporation, Armonk, NY, USA).

## Results

### Clinicopathological characteristics and their association with the immune cell score

The clinicopathological parameters and their relationships to the ICSs are shown in Table 1. We found no significant association between the ICS and any of the clinicopathological parameters.

### Prognostic impact of ICS on survival

Regarding the whole study group, the median follow-up time was 14 months. The estimated median OS was 20 months (95% CI 16.3–24.1). The 1-year, 3-year, and 5-year DSS rates were 72.0%, 27.5%, and 19.5%, respectively; the respective OS rates were 70.0%, 25.3%, and 17.9%.

**Fig. 2:**
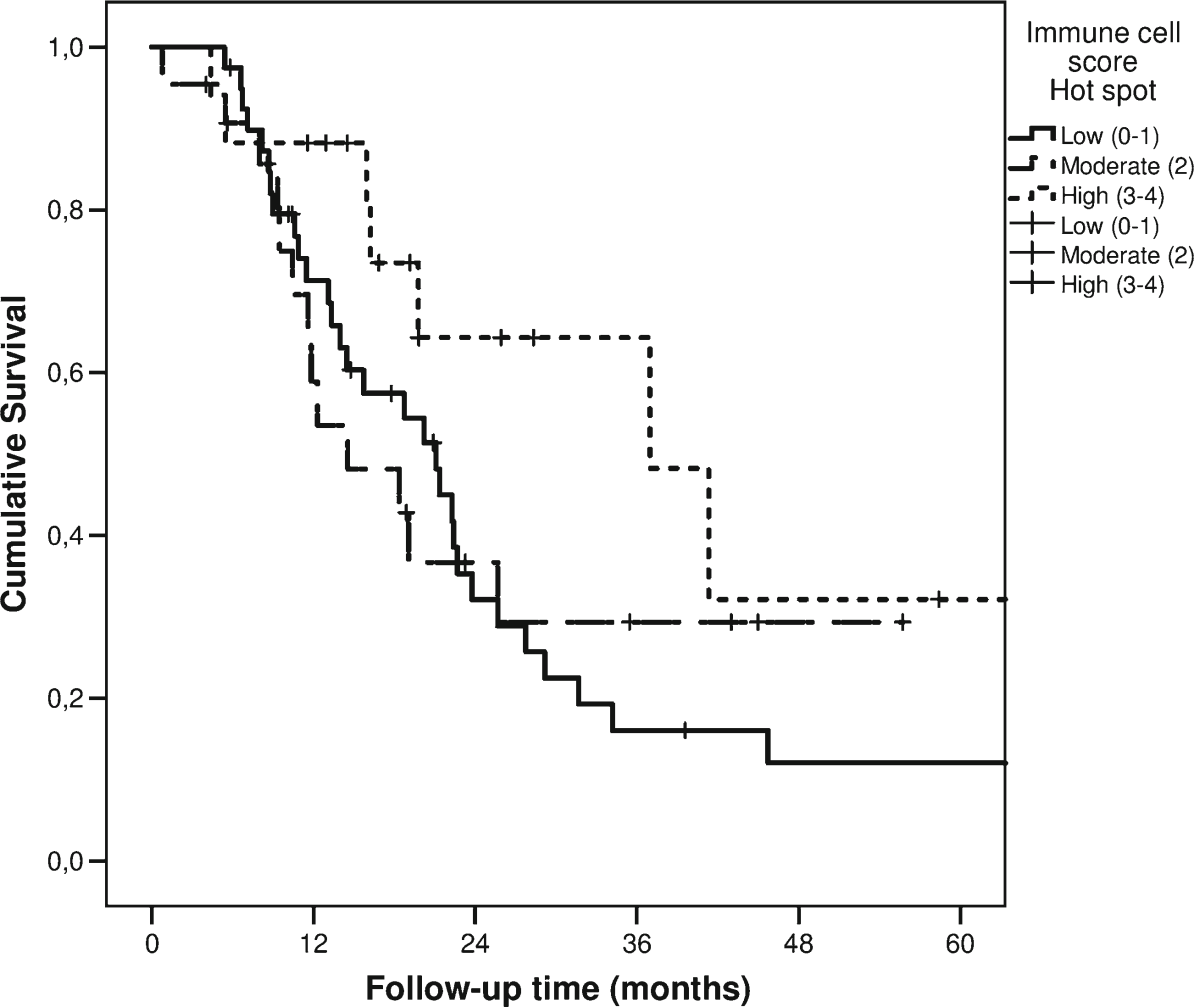
Disease-specific survival of patients with pancreatic ductal adenocarcinoma, stratified by low, moderate, and high immune cell scores, determined in hotspots

For the hotspot ICS analysis, the median DSS times for the low (ICS 0–1), moderate (ICS 2), and high (ICS 3–4) groups were 21 (95% CI 13.7–28.5), 15 (95% CI 5.4–23.7), and 37 months (95% CI 14.5–59.4), respectively. The 5-year DSS rates for the low, moderate, and high ICS groups were 12.1%, 29.4%, and 32.2%, respectively (*p* = 0.150; Fig. 2**)**. The median OS times for the low, moderate, and high ICS groups were 21 months (95% CI 13.7–28.5), 12 months (95% CI 7.9–16.7), and 37 months (95% CI 14.6–59.3), respectively. The 5-year OS rates for the low, moderate, and high ICS groups were 12.1%, 26.3%, and 26.8% (*p* = 0.193).

**Fig. 3:**
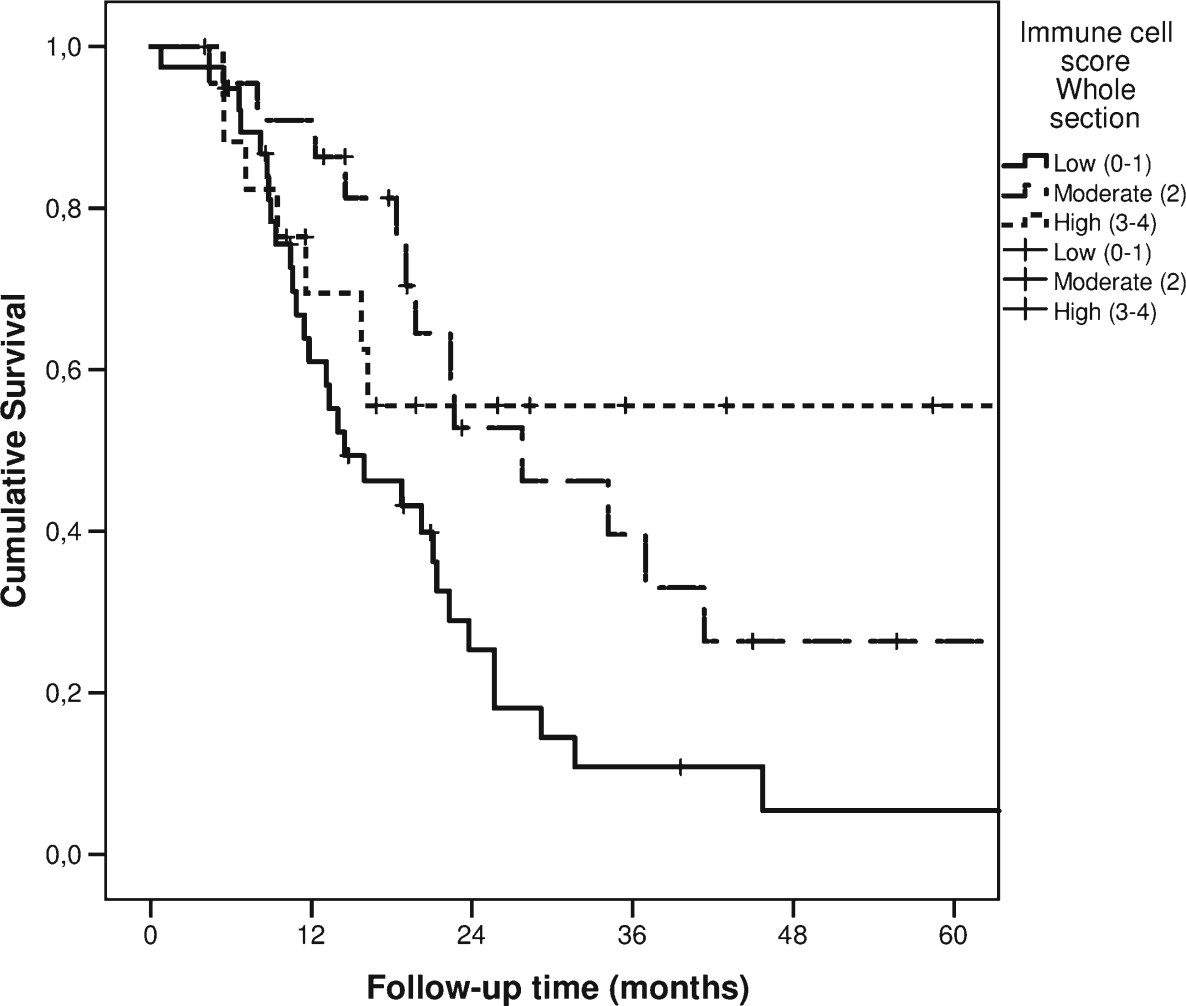
Disease-specific survival of patients with pancreatic ductal adenocarcinoma, stratified by low, moderate, and high immune cell scores, determined on whole tissue sections

The whole-section ICS analysis showed that the median DSS times for the low and moderate ICS groups were 14 (95% CI 7.1–21.9) and 28 (95% CI 13.3–42.2), respectively. A median DSS time was not reached in the high ICS group. The 5-year DSS rates for the low, moderate, and high ICS groups were 5.4%, 26.4%, and 55.6%, respectively (*p* = 0.020; Fig. 3). The median OS times for the low, moderate, and high ICS groups were 14 months (95% CI 10.4–18.5), 28 months (95% CI 13.3–42.2), and 26 months (95% CI 8.9–42.9), respectively. The 5-year OS rate for the low, moderate, and high ICS groups were 5.3%, 26.4%, and 43.8%, respectively (*p* = 0.030).

### Comparison of the measurement results (hotspots vs. whole sections)

We found that, when whole tissue sections were used to determine the number of immune cells, the ICS could predict patient survival (Fig. 3; Table 2). In contrast, the hotspot technique showed a similar trend but failed to show a significant association between the ICS and survival (Fig. 2; Table 2).

**Table 2:** Hazard ratios (HRs) with 95% confidence intervals (CI) of disease-specific and overall mortality of pancreatic ductal adenocarcinoma patients with low (0–1), moderate (2), and high (3–4) immune reaction based on immune cell score. Results based on whole sections and hotspots are presented separately

	Number of patients	Immune cell score 0–1 HR (95% CI)	Immune cell score 2 HR (95% CI)	Immune cell score 3–4 HR (95% CI)
Whole sections				
Disease-specific mortality				
All patients (crude)	79	1.00 (Reference)	0.48 (0.25–0.92)	0.41 (0.18–0.94)
All patients (adjusted)*	79	1.00 (Reference)	0.45 (0.21–0.95)	0.22 (0.08–0.60)
Overall mortality				
All patients (crude)	79	1.00 (Reference)	0.47 (0.25–0.89)	0.51 (0.24–1.07)
All patients (adjusted)*	79	1.00 (Reference)	0.42 (0.20–0.88)	0.27 (0.11–0.67)
Hotspots				
Disease-specific mortality				
All patients (crude)	79	1.00 (Reference)	0.92 (0.48–1.77)	0.45 (0.20–1.03)
All patients (adjusted)*	79	1.00 (Reference)	1.10 (0.52–2.34)	0.64 (0.26–1.58)
Overall mortality				
All patients (crude)	79	1.00 (Reference)	1.06 (0.57–1.98)	0.52 (0.24–1.13)
All patients (adjusted)*	79	1.00 (Reference)	1.41 (0.69–2.89)	0.70 (0.30–1.66)

*Adjusted for sex, age, tumour stage (TNM 8th edition), grade of differentiation, perineural invasion, radicality of resection (R0/R1)

Table 2 shows the Cox regression results from the unadjusted (crude) and adjusted models. The DSS and OS were analysed with ICSs determined with either the whole-section or hotspot technique. Results were similar when the models used the 7th edition of the UICC/AJCC TNM categories.

**Fig. 4:**
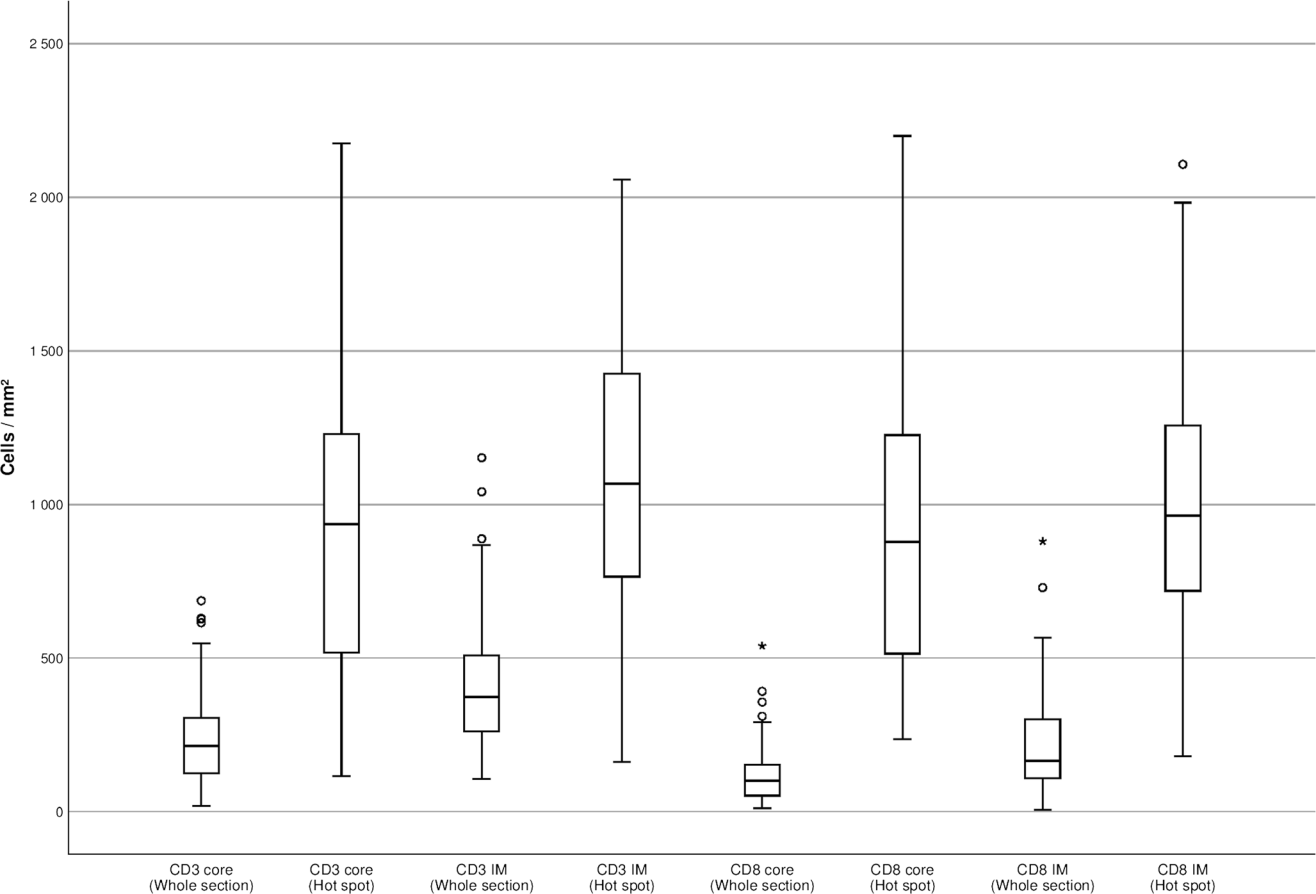
CD3^+^ and CD8^+^ T cell densities in the tumour core and invasive margin, presented separately for hotspots and whole tissue sections

Table 3 shows the unadjusted (crude) impact of each ICS component. The association between T cell densities and survival was stronger with the whole-section technique than with the hotspot technique. When we compared the numbers of immune cells between whole sections and hotspots, we found that the median number of immune cells per square millimetre was significantly higher in hotspots than in whole tissue sections. In particular, both the range and the interquartile range of immune cell counts determined with the hotspot technique were significantly larger than the ranges of the average immune cell densities observed in whole tissue sections (Fig. 4).

**Table 3:** Non-adjusted hazard ratios (HRs) with 95% confidence intervals (CI) of disease-specific and overall mortality of pancreatic ductal adenocarcinoma patients based on number of T cells (low and high). Results of whole sections and hotspots are presented separately

	Number of patients	Low HR (95% CI)	High HR (95% CI)
Whole sections			
Disease-specific mortality			
CD3 core (crude)	79	1.00 (Reference)	0.35 (0.13–0.98)
CD3 front (crude)	79	1.00 (Reference)	0.18 (0.39–1.20)
CD8 core (crude)	79	1.00 (Reference)	0.83 (0.47–1.46)
CD8 front (crude)	79	1.00 (Reference)	0.46 (0.26–0.81)
Overall mortality			
CD3 core (crude)	79	1.00 (Reference)	0.42 (0.17–1.06)
CD3 front (crude)	79	1.00 (Reference)	0.72 (0.42–1.23)
CD8 core (crude)	79	1.00 (Reference)	0.88 (0.51–1.52)
CD8 front (crude)	79	1.00 (Reference)	0.52 (0.30–0.90)
Hotspots			
Disease-specific mortality			
CD3 core (crude)	79	1.00 (Reference)	0.67 (0.36–1.23)
CD3 front (crude)	79	1.00 (Reference)	0.56 (0.29–1.08)
CD8 core (crude)	79	1.00 (Reference)	0.89 (0.47–1.70)
CD8 front (crude)	79	1.00 (Reference)	0.80 (0.46–1.42)
Overall mortality			
CD3 core (crude)	79	1.00 (Reference)	0.74 (0.41–1.32)
CD3 front (crude)	79	1.00 (Reference)	0.65 (0.35–1.19)
CD8 core (crude)	70	1.00 (Reference)	0.91 (0.49–1.71)
CD8 front (crude)	70	1.00 (Reference)	0.92 (0.53–1.59)

## Discussion

This study confirmed the significance of ICS as a prognostic factor in pancreatic cancer. We also found visible differences between the TMA-like hotspot and the whole-section techniques. Our analyses suggested that the whole-section technique was superior to the TMA-like hotspot technique. The whole-section technique indicated that the immune response had a significant impact on survival. With the hotspot technique, a similar trend was observed, but without statistical significance.

The association between the immune microenvironment of the tumour and the survival of patients with cancer had been well documented for PDAC [5, 21–23]. This association was also clearly shown in the present study. ICS was developed based on this association, combined with the notion of bringing together the prognostic value of several immune cell populations in different locations to obtain better prognostic value [1, 11, 18]. This study showed that the ICS determined with the whole-section technique predicted patient survival better than the ICS determined with the hotspot technique. However, the density of immune cells determined with the hotspot technique turned out to be significantly higher in this study compared to the previous study, despite the fact that we used hotspot sizes identical to the punch size used for TMA in the original ICS study [18]. This difference might be explained by the changes in immune cell densities from one level of section to the next. Moreover, in the TMA technique, small punches from hotspot areas are used to obtain a picture of the characteristics of the whole tumour. The inaccuracy of manual tissue punching makes it impossible to achieve consistency in selecting the most representative part of a tumour for TMA. In contrast, greater consistency can be achieved when the target area is defined from scans of the whole tissue section. Consequently, the TMA technique is more likely to show lower immune cell densities than the hotspot technique. In addition, tears in the TMA tissue sections can cause problems in estimating the sizes of analysed areas.

Tissue punches represent a small part of the whole tumour area. Alternatively, the development of image analysis has made it possible to count different cell populations rapidly and reliably in the whole tumour area [24]. In this study, the reliability of the whole tissue section technique over the hotspot technique was evidenced by the smaller variability between cases and the better performance in predicting survival. In future, the TMA technique will continue to play a role in research, when investigating specific characteristics in a large number of tissue samples. However, because the whole-section analysis is rapidly becoming easier, it may become the gold standard for estimating immune status in future, as evidenced by, e.g., the Immunoscore® method applied in colorectal cancer [11].

The mechanism that underlies the effect of immune cell infiltration on patient survival has not been fully established. Jamieson et al. showed that a large amount of immune cell infiltrate was associated with factors related to less malignancy, smaller tumour size, no lymph node metastases or intravenous invasion, and a lower stage of pancreatic cancer [23]. It has been suggested that the co-expression of CD4^+^ and CD8^+^ cells could serve as a prognostic factor in pancreatic cancer [5, 18, 22]. Immune cells might function, in the early stages of carcinogenesis, by preventing the spread of tumour cells to distant sites. Accordingly, pancreatic cancers with low amounts of immune cell infiltrate, as observed in our material, might have more invasive and metastatic potential, due to a greater ability to evade the immune system. Carstens et al. showed that effective cytotoxic T cell function appeared to require a location close to the cancer cells [25]. It was previously suggested that strong desmoplasia must play an important role in pancreatic cancer immune evasion. However, a recent study challenged this conviction by showing that desmoplasia did not impair T cell infiltration into pancreatic cancer tissue [25].

Given the fact that the impact of novel immunomodulating therapies appears to depend on the state of the local host immune system, it is important to continue developing tools for measuring that state. Our results indicated that the ICS could provide important additional information to traditional methods of TNM-staging in PDAC. However, before the ICS can be used in routine clinical applications, future studies are needed to provide a methodological validation of optimal cut-off values, based on several study populations [24]. Moreover, the value of this method must be validated in a prospective, multi-institutional setting.

This study had some limitations. The number of patients was relatively small, which limited the size of ICS subgroups, and resulted in low statistical power. Moreover, the follow-up time was relatively short, a common problem in PDAC, due to high mortality. We could not validate the previously determined cut-off values in this population because the immune cell densities varied significantly, due to differences between the manual TMA and hotspot techniques and due to technical differences between laboratories [18]. The main strength of the present study was the use of a consecutive patient series from a single geographical area of Northern Finland; thus, we could avoid a selection bias.

In conclusion, we confirmed that the ICS could predict post-surgical survival in patients with PDAC. In addition, our results suggested that the ICS determined in whole tissue sections provided higher prognostic value than the ICS determined in hotspots.

## Abbreviations

ICS: Immune cell score
PDAC: Pancreatic adenocarcinoma
DSS: Disease-specific survival
OS: Overall survival
TMA: Tissue microarray
